# Sustainable reduction of antibiotic-induced antimicrobial resistance (ARena) in German ambulatory care: study protocol of a cluster randomised trial

**DOI:** 10.1186/s13012-018-0722-0

**Published:** 2018-02-05

**Authors:** Martina Kamradt, Petra Kaufmann-Kolle, Edith Andres, Tonia Brand, Anja Klingenberg, Katharina Glassen, Regina Poß-Doering, Lorenz Uhlmann, Katharina Hees, Dorothea Weber, Andreas Gutscher, Veit Wambach, Joachim Szecsenyi, Michel Wensing

**Affiliations:** 1 Department of General Practice and Health Services Research, University Hospital Heidelberg, Im Neuenheimer Feld 130.3, 69120 Heidelberg, Germany; 2 aQua Institut GmbH, Maschmuehlenweg 8-10, 37073 Goettingen, Germany; 3 Department of Medical Biometry, Institute of Medical Biometry and Informatics, University Hospital Heidelberg, Im Neuenheimer Feld 130.3, 69120 Heidelberg, Germany; 4 Agentur deutscher Arztnetze, Chausseestraße 119b, 10115 Berlin, Germany

**Keywords:** Antibiotics, Inappropriate prescribing, Infection, Antimicrobial resistance, Ambulatory care, Randomised trial, Implementation science, Germany

## Abstract

**Background:**

Despite many initiatives to enhance the rational use of antibiotics, there remains substantial room for improvement. The overall aim of this study is to optimise the appropriate use of antibiotics in German ambulatory care in patients with acute non-complicated infections (respiratory tract infections, such as bronchitis, sinusitis, tonsillitis and otitis media), community-acquired pneumonia and non-complicated cystitis, in order to counter the advancing antimicrobial resistance development.

**Methods:**

A three-armed cluster randomised trial will be conducted in 14 practice networks in two German federal states (Bavaria and North Rhine-Westphalia) and an added cohort that reflects standard care. The trial is accompanied by a process evaluation.

Each arm will receive a different set of implementation strategies. Arm A receives a standard set, comprising of e-learning on communication with patients and quality circles with data-based feedback for physicians, information campaigns for the public, patient information material and performance-based additional reimbursement. Arm B receives this standard set plus e-learning on communication with patients and quality circles with data-based feedback tailored for non-physician health professionals of the practice team and information material for tablet computers (culture sensitive). Arm C receives the standard set as well as a computerised decision support system and quality circles in local multidisciplinary groups.

The study aims to recruit 193 practices which will provide data on 23,934 patients each year (47,867 patients in total).

The outcome evaluation is based on claims data and refers to established indicators of the European Surveillance of Antimicrobial Consumption Network (ESAC-Net). Primary and secondary outcomes relate to prescribing of antibiotics, which will be analysed in multivariate regression models. The process evaluation is based on interviews with surveys among physicians, non-physician health professionals of the practice team and stakeholders. A patient survey is conducted in one of the study arms. Interview data will be qualitatively analysed using thematic framework analysis. Survey data of physicians, non-physician health professionals of the practice team and patients will use descriptive and exploratory statistics for analysis.

**Discussion:**

The ARena trial will examine the effectiveness of large scale implementation strategies and explore their delivery in routine practice.

**Trial registration:**

ISRCTN, ISRCTN58150046. Registered 24 August 2017.

**Electronic supplementary material:**

The online version of this article (10.1186/s13012-018-0722-0) contains supplementary material, which is available to authorized users.

## Background

Antimicrobial resistance remains high on the health agenda. In Germany, a national policy agenda has reinforced policies to restrain antibiotic prescribing in medicine [1]. Despite decades of scientific research on rational use of antibiotics, and subsequent development of evidence-based practice guidelines [2], there remains a substantial room for improvement. For instance, data on patients with lower respiratory tract infections in German general practice showed that antibiotics were prescribed in 41% of consultations, of which 52% were congruent with guideline recommendations [3]. Strategies to optimise antibiotic prescribing can be effective. A cluster randomised trial in 259 practices in six European countries found that continuing education for physicians reduced their antibiotic prescribing by about 50% (from about 80 to 40%) in patients with respiratory tract infections [4]. However, it is unclear whether these impacts can also be achieved in large scale programs. The focus in the study described here is on scaling up existing implementation strategies to achieve sustainable and large-scale uptake of recommended use of antibiotics in ambulatory care in Germany.

In order to address a wide range of barriers for implementation, the increased rational use of antibiotics requires a complex implementation strategy––an intervention with multiple components interacting with each other. Based on relevant published research, which was identified through a literature review, and experience in quality improvement programs [5, 6], comprehensive implementation programs were developed in close collaboration with participating practice networks as key stakeholders. These programs address physician knowledge and attitudes about the use of antibiotics as well as the ambulatory care team, patient experiences and reimbursement. In the planned study, the effectiveness of these implementation programs will be examined.

A process evaluation will be conducted to understand the mechanisms underlying the effectiveness and to ensure generalizability of intervention effectiveness [7, 8]. Therefore, the process evaluation will explore which components of the programs and which contextual factors contribute to the effects. This knowledge is essential for building an evidence base that informs policy and practice [9]. The process evaluation will focus on the role of networks of ambulatory practices in Germany (‘Arztnetze/Praxisnetze’), in which the ARena project is embedded. These practice networks organise quality improvement and additional reimbursement for specific activities for participating ambulatory practices. Exploring the impact of those networks on antibiotic prescribing is of particular interest. The networks may enhance the contagion of new ideas and practices through various mechanisms of social influence between participating healthcare providers and thus enhance the contagion of knowledge and practices [10]. Reducing antimicrobial resistance is only possible if the large majority of physicians join efforts to reduce antibiotic prescribing. The context of a sustained network may influence physicians’ willingness to cooperate with programs such as ARena because the repeated interaction in the network enhances mutual trust and facilitates the exclusion of non-cooperators [11].

The planned study has three central aims: (a) to determine and compare the effectiveness of three implementation programs to optimise antibiotic prescribing in ambulatory care, (b) to compare the outcomes of the three implementation programs with standard care and (c) to provide insight into the fidelity of the implementation programs, their potential working mechanisms and the impact of health system-related factors on outcomes.

## Methods/design

### Study design

**Fig. 1:**
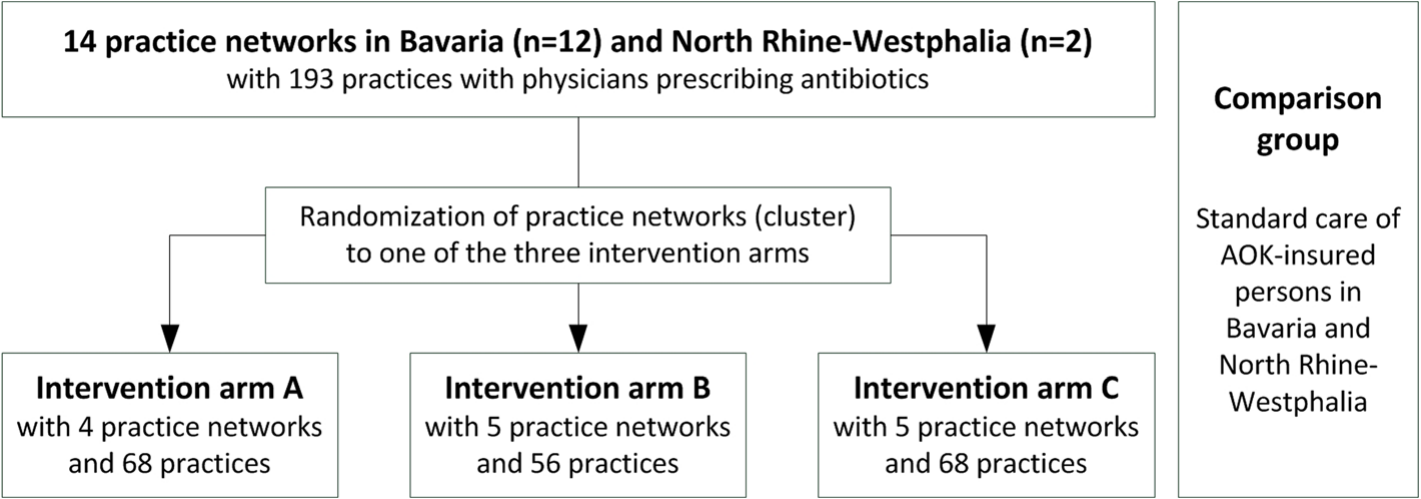
ARena study design with three intervention arms and comparison group

This three-armed (non-blinded) cluster randomised trial consists of different components which aim to achieve sustainable and large-scale uptake of recommended use of antibiotics in ambulatory care in Germany. Each of the three arms will receive a different set of components. The components are based on published research and experiences in improvement programs. Figure 1 presents the study design.

**Fig. 2:**
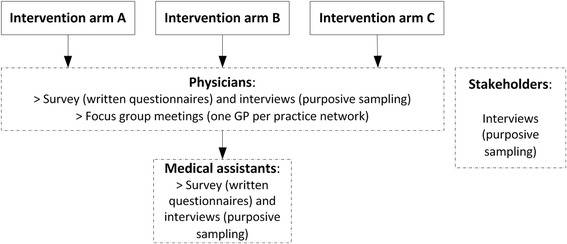
Process evaluation design of the ARena study

The study is planned for 30 months, with an intervention period of 24 months, and split into two parts of evaluation: (a) an *outcome evaluation* based on claims data and (b) a *process evaluation*. The process evaluation will focus on physicians allocated to one of the three intervention arms, non-physician health professionals of the practice team in intervention arm B, as well as stakeholders (see Fig. 2). In Germany, beside physicians, only one non-physician health professional (‘Medizinische Fachangestellte’) is involved in ambulatory care. This role is comparable to medical assistants in the USA [12]. Additionally, a *patient survey* will be conducted in intervention arm B.

The ARena study is approved by the ethics committee of the Medical Faculty of the University of Heidelberg (reference number S-353/2017).

### Implementation strategies

The implementation strategies will be gradually applied during the intervention period of the study. Intervention group A receives a conventional improvement program with five components targeting physicians and patients. Intervention group B receives the conventional program as well as additional components, which target medical assistants and patients. Intervention group C receives the conventional program and a different set of additional components which target physicians as well as other healthcare providers, like pharmacist and representatives of hospitals or nursing homes. In total, the implementation programs consist of 10 intervention components (Table 1) and take the concept of blended learning into consideration. The specific components are briefly described in this section and characterised in more details according to the TIDieR checklist [13] in Additional file 1.

**Table 1:** Overview of intervention components

	Intervention arm		
Intervention components	A	B	C
1) E-learning for physicians	x	x	x
2) Quality circles with data-based feedback for physicians	x	x	x
3) Information campaigns for the public	x	x	x
4) Information campaigns for patients in respective practices	x	x	x
5) Performance-based additional reimbursement (P4P)	x	x	x
6) E-learning for medical assistants		x	
7) Quality circles with data-based feedback for medical assistants		x	
8) Information material available on tablet computers		x	
9) Computerised decision support system (CDSS)			x
10) Quality circles in local multidisciplinary groups			x

Intervention group A (multifaceted intervention program for physicians and patients):

1. E-learning on communication with patients for physicians: Patient-centred communication skills are trained, using videotaped consultations, checklists for use in consultations and patient education material. The focus is on exploration of patient expectations, delayed prescription, if necessary, and shared decision making.
2. Quality circles with data-based feedback for physicians: Four moderated meetings of physicians spread over a period of 2 years, using practice-specific feedback on prescriptions as well as evidence-based information on antimicrobial resistance and prescribing of antibiotics as input.
3. Information campaigns for the public: The information campaign includes social media (Facebook, Twitter, YouTube), explanatory videos, expert interviews, radio and print material. Patient information is provided online (arena-info.org; arena-info.de), using lay language and a user-responsive design. Local healthcare providers are involved, as far as possible. Patient leaflets, which can be individualised to practices, are available in various languages. The information for the public relates to relevant other campaigns on antimicrobial resistance and use of antibiotics, such as the European antibiotics day (18th November––every year).
4. Patient information material: Posters and leaflets are provided to deliver information on antibiotic prescribing to patients with acute non-complicated infections, mainly using existing material from established organisations.
5. Performance-based additional reimbursement: Practices with relatively high numbers of patients with acute respiratory symptoms, bronchitis, tonsillitis, sinusitis or otitis media without antibiotic prescription receive additional reimbursement. The algorithm for the performance-based payment, which will be developed, considers the empirical distribution among practices and the available budget.

Intervention group B (multifaceted intervention program for non-physician health professionals of the practice team and patients):

Components 1–5 plus:

6) E-learning on communication with patients for medical assistants: Medical assistants (‘Medizinische Fachangestellte’) receive training, which is focused on communication with patients and practice organisation.
7) Quality circles with data-based feedback for medical assistants: Four moderated meetings of medical assistants spread over a period of 2 years, using practice-specific feedback on prescriptions and patient surveys.
8) Information material for tablet computers: A tablet computer (culture sensitive tailoring: English, Turkish, Russian, Arabic) is provided to deliver information on antibiotic prescribing to patients with acute non-complicated infections, mainly using existing material from established organisations.

Intervention group C (multifaceted intervention program for physicians with additional strategies):

Components 1–5 plus:

9) Computerised decision support system (CDSS): A CDSS is installed in the practice information system. After coding of a relevant diagnosis or antibiotics in the practice system, the CDSS generates a pop-up with a recommendation on prescription of antibiotics based on evidence-based clinical guidelines. The tool is provided by different software providers.
10) Quality circles in multidisciplinary groups: Representatives of local healthcare providers (such as ambulatory physicians, hospitals, nursing homes, home nursing, pharmacists) are invited to multidisciplinary, cross-sectoral quality circles. The emphasis of the meetings is on providing information and education on antimicrobial resistance and antibiotic prescribing and to coordinate a regional joint course of action for the rational use of antibiotics.

### Study population

Fourteen practice networks in two German federal states (Bavaria and North Rhine-Westphalia) are recruited for participation in the ARena project. For administrative reasons, the study will focus on patients insured by AOK health insurance, which are registered within a specific program for healthcare delivery (‘Besondere Versorgung’ as defined by German law § 140a SGB V a.F. bzw. § 140a Abs. 1. S. 2 Alt. 1 SGB V n.F). Compared to patients insured by other German health insurances, AOK-insured patients have somewhat lower income, less education, higher probability of migration background and lower self-reported health status [14]. On average, 35% of the population within the geographic reach of the participating practice networks in Bavaria are insured by AOK health insurance. At baseline, approximately 40,000 patients with AOK health insurance are registered in 193 participating ambulatory care practices in these 14 networks. In total, there are 304 eligible ambulatory care practices in these 14 networks. The patient population for the intervention arms will comprise patients who attended ambulatory practices for the following reasons: acute non-complicated infections of the respiratory tract (bronchitis, sinusitis, tonsillitis, otitis media), non-complicated cystitis and community-acquired pneumonia (CAP). The diagnoses will be based on physician-recorded ICD codes in administrative data, which are provided for reimbursement and lumped in 3-month periods.

### Inclusion criteria

In order to be able to fill in the data collection tools used within the process evaluation (e.g. questionnaires), all physicians, medical assistants, stakeholders and patients, participating in the patient survey, should have written and spoken German language skills and have to be 18 years of age or older. Additionally, written informed consent is a prerequisite for participation in the study.

#### Ambulatory practices and healthcare providers

Ambulatory practices need to be enrolled in one of the 14 participating practice networks in Bavaria and North Rhine-Westphalia to be eligible for participation. In addition, physicians need to participate in one of the following medical specialist groups: general practitioner (Facharzt für Allgemeinmedizin, GP), internists, gynaecologist, ENT specialist, urologist, pulmonary specialist or paediatrician (Additional file 2: Table S1). Out of these eligible ambulatory practices, physicians specialised in general practice or internal medicine and functioning as GP will be included in the outcome evaluation. All physicians, who participate in one of the intervention arms, will be invited to take part in the process evaluation. Additionally, GPs included in the outcome evaluation will have the opportunity to participate in focus group meetings.

Medical assistants employed at one of the eligible and participating ambulatory practices, which are allocated to intervention arm B, are eligible for study participation and will be invited to take part in the process evaluation, as well.

#### Patients

Eligible are patients with a diagnosis of an acute non-complicated infection of the respiratory tract (bronchitis, sinusitis, tonsillitis, otitis media), acute non-complicated cystitis or CAP who are insured by AOK health insurance in Bavaria or North Rhine-Westphalia. Eligible patients of ambulatory practices in intervention arm B, which are insured by the AOK health insurance in Bavaria, will be invited to participate in a patient survey.

### Exclusion criteria

No additional exclusion criteria for participants are defined. Patients, stakeholders, ambulatory practices and healthcare providers who do not fulfil the specific inclusion criteria in each part of the study (outcome evaluation, process evaluation and patient survey) will be excluded.

### Study procedures

The primary as well as a few secondary outcomes are based on pseudonymized *claims data* (§§ 295, 300 SGB V). Claims data are based on billing data of physicians––like medical prescriptions, diagnoses (ICD-10 codes) and medical service according to the uniform reimbursement scale (‘Einheitlicher Bewertungsmaßstab’) for medical services. Additionally, routine data of statutory health insurance will be used: (a) hospital and ambulatory treatments (as defined by German law §301 SGB V), (b) service of statutory nursing care insurance (as defined by § 105 SGB IX) and (c) basic claims data (‘Stammdaten’, as defined by § 284 SGB V).

The process evaluation is based on surveys and interviews with healthcare providers and stakeholders. Written questionnaires will be given to physicians and, in intervention arm B also to medical assistants, three times during the intervention period. Face-to-face and telephone interviews with physicians, medical assistants and stakeholders will be conducted after the first winter period. The interviews will provide additional insight into perceived impact of intervention components and further explore contextual factors focusing on impact and structures of information exchange in practice networks.

Additionally, focus groups will be used to gain feedback from members of the targeted group at all stages (see Fig. 2) of the planning, implementation and follow-up of ARena interventions and to learn from their experiences and optimise the interventions.

The patient survey will include patients with acute non-complicated infections in participating practices in study arm B. Eligible patients will be identified by their physician (insured by AOK health insurance in Bavaria and diagnosed with an acute non-complicated infection of the respiratory tract, non-complicated cystitis or CAP) and asked to take part in the patient survey by filling in the anonymous questionnaire.

For a detailed recruitment description of study participants, see Additional file 2: Table S2.

#### Financial compensation for study participation

In total, three billing items will be used to reimburse the patient and study-related extra effort. All participating physicians will receive 6€ and medical assistants 5€ per eligible patient and quarter year. Additionally, physicians in intervention group C using the CDSS will receive 2.81€ per eligible patient and quarter year.

Physicians, medical assistants and stakeholders who participate in the interviews within the process evaluation will receive a reimbursement of 50€ each.

### Randomisation procedure

The allocation to the three arms of the study is done by the study statisticians, using a computer program, and concealed from all others in the project. The 14 practice networks are allocated to one of the three intervention arms under the constraints that the arms contain a similar number of practices (not more than one practice difference) based on an estimated participation rate of practices within the networks and that intervention arm A contains 4 practice networks and intervention arms B and C each 5 practice networks. From a total of 34,650 possible allocation outcomes, 112 meet this requirement, from which a computer program randomly picks one.

### Recommended clinical practice

Recommendations for antibiotic prescribing are derived from several German high-quality, evidence-based clinical guidelines, most of which were developed by the German College of General Practitioners and Family Physicians (DEGAM) [2, 15]. All guidelines used are systematically developed, evidence- and/or consensus-based and conform with the quality standards for guidelines in Germany [16]. In short, they recommend restrained prescription of antibiotics, particularly in healthy patients with acute non-complicated symptoms. If antibiotics are used, recommended ones should be preferred instead of broad-spectrum antibiotics, like quinolones, for example.

### Primary and secondary outcome measures

The primary as well as the secondary outcomes of the claims data based evaluation are defined and operationalized based on previous research in Germany [17]. Pseudonymized claims data (§§ 295, 300 SGB V) are used to construct different European Surveillance of Antimicrobial Consumption Network (ESAC-Net) indicators [18, 19] measuring the primary and secondary outcomes.

The primary outcome refers to the following index indicator, based on different single ESAC-Net indicators:

1. Percentage of patients with acute non-complicated infections who are treated with antibiotics.

Secondary outcomes refer to the following (ESAC-Net) indicators:

2. The use of antibiotics in ambulatory care in defined daily dose (DDD) per 1000 residents (respectively insured persons) per day and region
3. Percentage of DDD of broad-spectrum quinolones of all used antibiotics
4. Percentage of DDD of broad-spectrum cephalosporins (3rd and 4th generation) of all used antibiotics
5. Percentage of patients (18–75 years) with acute bronchitis, patients (> 18 years) with sinusitis, patients (> 2 years) with otitis media and patients (> 1 year) with upper respiratory tract infections/tonsillitis with a prescription of (a) recommended antibiotics, if necessary at all, and (b) less broad-spectrum antibiotics like quinolones
6. Percentage of women (> 18 years) with a diagnosis of non-complicated cystitis and a prescription of (a) antibiotics, (b) recommended antibiotics and (c) less broad-spectrum antibiotics like quinolones
7. Percentage of patients (18–65 years) with community-acquired pneumonia and a prescription of (a) antibiotics, (b) recommended antibiotics, (c) less broad-spectrum antibiotics like quinolones (> 16 years) and (d) less broad-spectrum antibiotics like cephalosporins or macrolides (> 16 years)
8. Percentage of patients with acute non-complicated infections who use medical emergency service
9. Percentage of patients with community-acquired pneumonia and hospitalisation

The process evaluation will use a mix of measures to assess the fidelity of the implementation programs, the potential working mechanisms and the impact of health system-related factors on outcomes. The impact of participation in a practice network on implementation of new ideas and practices regarding antibiotic prescribing is of particular interest. Various social mechanisms influence the spread of new attitudes and behaviours, as well as the motivation to change the behaviour [10]. In the exploration, some emphasis will be put on the potential impact of the networks in which the physicians are embedded. Therefore, tailored questions for interviews and surveys are phrased, which cover (a) the uptake and perceived impact of intervention components by participants with a focus on handling patients with acute non-complicated infections, (b) the perceived impact of contextual factors, particularly those related to practice networks, and (c) perceptions of patients’ expectations regarding antibiotic prescribing. The construction of the survey is based on Ajzen’s [20] theory of planned behaviour. The survey items are conform with this theoretical framework of Ajzen [20] and are used to gain specific information about the impact of (a) each intervention component received, (b) contextual factors including structural conditions and organisation of patient care, legal terms and conditions (e.g. legal quality requirements, reimbursement of patient care by statutory health insurance) and participation in a practice network, and (c) perceptions of patients expectation regarding antibiotic prescribing on the behaviour of participating physicians and medical assistants. Items are scored on a 5-point Likert scale ranging from 1 (strongly disagree) to 5 (strongly agree). Interviews will add in-depth understanding of mechanism of action, particularly those related to practice networks, and explore uptake and possible adaption to daily practice of intervention components in more detail. Both surveys and semi-structured interviews include written questions concerning socio-demographic aspects like age, gender, years of working experience and characteristics of the working environment.

Focus group interviews will aim to discuss barriers in implementation of intervention components and to develop possibilities to overcome these barriers.

Patient survey will be used to gain feedback from patients concerning their knowledge and views on antibiotics and antibiotic prescribing, as well as their perception of different intervention components targeting patients. Therefore, a questionnaire will be used covering reason for consultation (complaints/illness), whether antibiotics are expected, requested and received (yes/no questions), as well as (with 4-point Likert scale) patient education and shared decision making on antibiotic prescribing, views on delayed antibiotic prescription, perceived study components, the role of non-physician health professionals of the practice team, general view on antibiotic prescribing in and general satisfaction with the practice. In addition, age, sex, nationality and education are recorded. The questionnaire used in the patient survey is based on published research [21, 22].

### Data collection and data management

#### Outcome evaluation

Primary and secondary outcomes, as well as patient characteristics, will be extracted from administrative data at the health insurers involved, so called claims data. This will be done each quarter year. These data provide the basis for the outcome evaluation and the written feedback for participating practices.

Patient characteristics documented from the administrative data include age, sex, years in insurance, insurance status (principal, family or retired member), patient participation in disease management programs (diabetes, breast cancer, asthma, coronary heart disease, COPD, chronic heart failure) and other additional care programs. In addition, prescribing physician identification, medical discipline and physician participation in disease management programs are recorded.

#### Process evaluation

Written, paper-based questionnaires will be mailed to participating physicians in study arms A, B and C, and in study arm B also to medical assistants. This will be done three times following their quality circle meetings by the study central office. Reminders will be sent out after 2 weeks to increase the response rate. The filled in questionnaire will be sent to the Department of General Practice and Health Services Research, University Hospital Heidelberg. Hence, the survey is pseudonymized, no identification of the participants’ identity will be possible.

The questionnaire will be pilot tested with a sample of four physicians, who are associated with the Department of General Practice and Health Services Research, University Hospital Heidelberg and two non-physician health professionals of the practice team.

Face-to-face and telephone interviews with a sample of 40 physicians and medical assistants as well as an additional sample of 10 stakeholders will be done by researchers. Each interview will take between 30 to 45 min and is conducted using a semi-structured interview guide. The interviews will be audio-recorded, transcribed verbatim and stored on a secured server of the University Hospital Heidelberg. Transcripts will be pseudonymized.

Focus group interviews with one representative of each practice network will be done every 3 months.

#### Patient survey

Sixty AOK-insured patients per participating practice in Bavaria in intervention arm B, who visit the practice with at least one of the relevant index diseases, will be invited to complete a written questionnaire after the consultation. The questionnaire is filled in anonymously by each patient and is collected in a sealed box in each practice. A time slot of maximum 3 months is defined for conducting the survey. Afterwards, the sealed box containing the questionnaires is sent back to the aQua-Institute for data analysis.

A pilot study including telephone interviews with patients will be conducted in advance to ensure that the questions are comprehensible from the patients’ point of view.

### Data analysis

#### Outcome evaluation

The primary objective of this study is to examine the change of the antibiotic prescription rate in targeted patients within three intervention arms and the comparison between the three intervention arms. The confirmatory analysis of the primary endpoint will be conducted on the basis of the Intention-To-Treat (ITT) population where all patients will be included in the analysis and assigned to the group they were randomised to.

To account for multiple testing and to assure a global significance level of 5%, a multiple test procedure for hierarchically ordered hypotheses will be applied. In the first stage, differences regarding the primary endpoint (prescription of antibiotics) before and after the intervention in intervention groups B and C are tested. To assure a significance level of 5% within the first level, the hypotheses for the pre- and post comparison will be tested at a local significance level of 5/2%. The second step includes the pre-and post comparison in intervention group A. The significance level of the rejected hypotheses from step one is taken (2.5% if only one hypothesis can be rejected, 5% if both hypotheses can be rejected). If significance is reached in intervention groups A and B, differences between groups A and B will be tested at a local significance level of 5/2%. Similarly, difference between groups A and C will be only tested if significance is reached for groups A and C in the steps 1 and 2. If the hypotheses in step 1 or 2 cannot be rejected, the group comparison will not be tested primarily.

In all stages described above, a logistic mixed effects model will be applied to assess the respective hypotheses regarding the primary endpoint. In the first and second stage, the time (before/after the intervention) is included as fixed effect. A random intercept will be included for patients and for practices. For the comparisons in the third stage, the intervention group will be included as fixed effect additionally. Furthermore, age and gender will be included as covariates. Finally, sensitivity analyses will be conducted using different populations (per protocol population where patients with major protocol violations are excluded and appropriate subgroups). Since all these analyses are based on claims data, missing values cannot be tracked.

Descriptive methods will be used for the analysis of the secondary outcomes, including the calculation of appropriate summary measures of the empirical distribution (mean, standard deviation, median, IQR, minimum and maximum for continuous variables and frequency in percentages for categorical variables). In addition, similar mixed models as described for the primary endpoint will be used. All calculated *p* values regarding secondary outcomes will have descriptive character only.

Furthermore, differences between the intervention groups and an untreated comparison group will be tested. The comparison group consists of claims data of patients insured by AOK health insurance in non-participating practices in Bavaria and North Rhine-Westphalia. Therefore, propensity score matching will be used to generate 14 virtual clusters of non-participating practices.

A detailed statistical analysis plan is written prior to the final analysis. SAS (SAS Institute Inc., Cary, NC, USA), version 9.4 or higher, is used to carry out the analyses.

### Process evaluation

Descriptive statistics will be used to characterise the study sample and to analyse the survey data of physicians and medical assistants according to intervention fidelity, e.g. adherence to different intervention components. Moreover, regression analysis will be used to gain information about the dependence between claims data based outcome measures and (a) the uptake and perceived impact of intervention components by participants, (b) the perceived impact of contextual factors, particularly those related to the practice networks, (c) perceptions of patients’ expectations regarding antibiotic prescribing.

The semi-structured interviews will be audio-taped and transcribed verbatim. The interview data will be qualitatively analysed using thematic framework analysis to classify and organise data according to key themes, concepts and predefined categories. The transcripts will be analysed and coded by researchers using qualitative data analysis software (e.g. Atlas.ti). Predefined categories of the framework of Flottorp et al. [23] will be used to identify determinants of practice regarding improvements in health professional practice concerning the appropriate use of antibiotics in acute non-complicated infections in ambulatory care.

Aims of the focus group discussions are (a) to understand opinions of the targeted groups of study participants, (b) to identify barriers in the implementation process and (c) to reflect consequences for a possible further development of the intervention. The duration of discussion per topic during the meetings will serve as parameter to identify key topics, main barriers and discussed solutions to overcome possible difficulties. Basis of this analysis will be structured manuals and audiotapes of each meeting.

Data triangulation will occur through an integrated approach where qualitative and quantitative data will be systematically incorporated. This will be done after all sets of data have already been analysed individually. This approach strengthens the analysis by taking a more holistic approach to analysing data and evaluating the study [24].

#### Patient survey

Descriptive statistics will be used to analyse the data collected through the patient survey. This will be done (a) in order to characterise the patient sample, (b) to show the frequencies of answers given to the questions (e.g. antibiotics expected vs. antibiotics received, information on antibiotics received, item response). Additionally, correlation and regression analysis will be done with data collected in the main surveys, e.g. in order to detect dependences between patients’ characteristics (age, sex, etc.) or their actual (index)-disease and patients’ experiences and expectations concerning antibiotic prescribing.

### Sample size calculation

The sample size calculation is based on the expectation to find an absolute reduction of the antibiotic prescription rate in targeted patients from 61 to 50% in intervention group A and from 61 to 45% in groups B and C. Thus, the effect is expected to be 5% higher in the enriched intervention groups B and C compared to intervention group A.

To control the overall type I error rate at 0.05 (two-sided), a hierarchical 3-step testing procedure will be applied. In the first step, the hypotheses of no difference between the pre- and post prescription rate in group B and C will be tested. If at least one of these hypotheses can be rejected, the hypothesis of no difference in the pre- and post rate in group A will be tested. If, additionally, this hypothesis can be rejected, the hypotheses of no difference between the post rates of groups A and B as well as groups B and C will be tested. The sample size is calculated based on chi-squared tests assuring a (global) power of 80% for the rejection of all the above described, hierarchically ordered hypotheses. Furthermore, to take the clustered structure into account, an intra-cluster correlation coefficient (ICC) for patients in practices of 0.05 and of 0.7 for cases clustered in patients is assumed. Together with the above described expected pre- and post prescription rates in the three groups, this results in a sample size of 23,933 patients in group A, 11,967 patients in group B and 11,967 patients in group C. With an average practice size of 80 patients per quarter this leads to 75 practices in group A, 38 practices in group B and 38 practices in group C.

## Discussion

The evidence base of large scale programs to reduce antibiotic prescribing in acute non-complicated infections in primary care is limited, so this project can provide an important contribution. Nevertheless, a number of limitations of the study should be acknowledged. The study is randomised, but the number of units for allocation (i.e. networks) is small, leaving room for bias. Primary and secondary outcomes are based on claims data, which have inherent limitations and strengths. Given the multifaceted programs, it will be difficult to separate out the impact of various program components. On the other hand, strong aspects of the program are its large scale and closeness to daily practice. The focus on practice networks and implementation outcomes, which will be explored in the process evaluation, adds value to the study. The analysis of components of the complex implementation programs, using the TIDier checklist, contributes to the transparency of the implementation programs.

## Additional files

Additional file 1: Intervention description according to TIDieR. (PDF 432 kb)

Additional file 2: Eligible medical specialist groups for participation in the ARena study (Table S1); description of the recruitment of participants in the ARena study (Table S2). (PDF 222 kb)

## Abbreviations

AOK: Allgemeine Ortskrankenkasse (health insurer)
aQua: Institute for Applied Quality Improvement and Research in Health Care
CAP: Community-acquired pneumonia
CDSS: Computerised decision support system
DDD: Defined daily dose
DEGAM: German College of General Practitioners and Family Physicians
ESAC-Net: European Surveillance of Antimicrobial Consumption Network
FG: Fachgruppe (medical specialist group)
GP: General practitioner
ICC: Intra-cluster correlation coefficient
ICD: International Classification of Diagnosis
ITT: Intention-to-treat
P4P: Pay for performance
PP: Per protocol
SFTP: Simple FILE Transfer Protocol
SGB V: Social Code Book V, Germany
