# Supplementary material for: Sustainable reduction of antibiotic-induced antimicrobial resistance (ARena) in German ambulatory care: study protocol of a cluster randomised trial
Source: Implement Sci. 2018 Feb 5;13:23. doi: 10.1186/s13012-018-0722-0 (PMC5800289; doi:10.1186/s13012-018-0722-0)
Supplement: Supplementary file 1 — Intervention description according to TIDieR. (PDF 432 kb) [file 13012_2018_722_MOESM1_ESM.pdf]

Additional file 1: Intervention description according to TIDieR

Component 1 E- learning on communication with patients for physicians

1 Short Name	E-learning for physicians to promote patient-centred communication.
2 Goal and rationale	The focus is on exploring patient expectations and shared decision-making in patients with non-complicated, acute infections, which are especially common in ambulatory practices and do not require the use of antibiotics as first choice treatment. The educational objective is to carry out a convincing physician-patient communication.
3 Materials	Video clips of re-enacted scenes in GP practices. Additional information as text / graphics.
4 Procedures	Various situations, typical for GP practices, are represented. In addition, the e-learning platform comprises background information, which can be displayed by the users, if required.<br>Control of learning success will be possible. Eligible employees of the aQua Institute will be able to track if the e-learning session was completed and generate personalized certificate.
5 Providers of intervention	The screenplay is produced at the Institute of General Medicine of the University of Rostock and implemented by the Society for Patient-centered Communication mbH (GPZK) in collaboration with a director and actors. The e-learning platform is established at the aQua Institute with the help of an external service provider.
6 Mode of delivery	Single intervention<br>All physicians who participate in ARena.
7 Location	The content is integrated into a Learning Management System (LMS). The learning platform will be accessible via a user-portal.<br>The learning contents in the learning platform are secured. Access for users can be provided via the specific user-portal. Users (learners) of the physician networks participating at ARena are captured electronically. The learning contents are structured as self-learning courses. The learning platform will be available under the domain „welearn.academy“.<br>The correct functioning of video contents is ensured by an early test. The learning contents are generated in HTML5 format using the SCORM standards.
8 Frequency	Each course lasts about 1 hours and can be completed in the 4th quarter of 2017.
9 Planned tailoring	No
10 Fidelity enhancement	

Component 2 Quality circles with data-based feedback for physicians

1 Short Name	Quality circles with data-based feedback for physicians.
2 Goal and rationale	Self-reflection by means of practice-specific, data-based feedback reports and evidence-based background information combined with discussion and exchange of experiences in quality circles together with other physicians.
3 Materials	Feedback reports are based on the claims data (diagnosis and prescription data) of one's own practice; checklists, patient information; slides for quality circle implementation
4 Procedures	Claims-data (§§295, 300 SGB V) are provided by the participating health insurance companies pseudonymized for practices and anonymized for patients. Participating practices have signed a written informed consent to use their practice specific data, which means that specific claims-data can be allocated to each participating practice. A SFTP (Secure FILE Transfer Protocol) will be used for transmission of data between health insurance and the aQua Institute. Afterwards data will be checked and analyzed using SPSS and evaluated at the operations identification number (BSNR) level.<br>Individualized reporting is enabled by a self-developed software. The benchmark (mean 25% best practices), own network, all networks, control supply (comparison group) are shown for comparison purposes. The background information is guideline- and evidence-based (structured literature research). Established indicators are used.
5 Providers of intervention	Physician, pharmacist, psychologist, programmer, medical documentation specialist
6 Mode of delivery	Individual and group intervention<br>All physicians who participate in ARena.
7 Location	The material will be sent by post (practice-specific feedback reports) or are provided in the login area of the project portal (samples, slides) (arena-info.de).
8 Frequency	4x ca. 2 hours in the quarters 2017q4, 2018q2 + q4, 2019q2
9 Planned tailoring	
10 Fidelity enhancement	

Component 3 Information campaigns for the public

1 Short Name	Information campaigns for the public.
2 Goal and rationale	The aim is to reach the public through various media and to raise awareness on the topic of "antibiotics / resistance development". Dissemination of core messages throughout societies.
3 Materials	Internet portal;<br>Posters & flyers for the waiting room;<br>non-pharmakological information / recommendations<br>Social media (Facebook, Twitter, You Tube);<br>Information in regional print, radio and TV media;<br>Short video clips of interviews with physicians<br>Topic-specific explanation videos via the portal<br>Cartoons which explain what antibiotics are, how they should be used or not used etc.
4 Procedures	Disseminated core messages are guideline-based and coordinated with the other consortium partners. Involved practice networks will use material from widely recognized institutions, as well as self-generated ones in order to create a „Corporate Identity“. All material are made available for the participating networks and practices via the ARena portal for free.
5 Providers of intervention	Physicians, pharmacists, psychologist, journalists, advertising experts.
6 Mode of delivery	Group intervention<br>In the regions of all ARena networks, but also nationally.
7 Location	Provision via ARena Portal and Facebook, Twitter, You Tube
8 Frequency	2018q1, 2018q4, 2019q1, 2019q2
9 Planned tailoring	
10 Fidelity enhancement	

Component 4 Patient information material

1 Short Name	Patient information material.
2 Goal and rationale	The information material focuses particularly on patients affected by infections and aim to raise awareness for the topic „antibiotics / resistance development”.
3 Materials	Handout / Info-Tips (guideline-based, culture-sensitive)<br>Green slip prescriptions (‘Grüne Rezepte’, physician recommended, but not subject to prescription medication, usually not reimbursed by health insurer)
4 Procedures	The core messages are guideline-based and developed in agreement with the other consortium partners. The material used are from recognized institutions, as well as self-generated ones in order to create a „Corporate Identity “. All material are made available for the participating networks and practices via the ARena portal for free.
5 Providers of intervention	Physician, pharmacist, psychologist, journalist, advertising expert.
6 Mode of delivery	Group intervention<br>Some of the material is also available via the portal.
7 Location	Provision of material for the practices via the ARena portal
8 Frequency	2017q4, 2018q1+q4, 2019q1+q2
9 Planned tailoring	
10 Fidelity enhancement	

Component 5 Performance-based additional reimbursement

1 Short Name	P4P, performance-based additional reimbursement
2 Goal and rationale	Performance-based additional reimbursement to support rational antibiotics prescription like proposed.
3 Materials	claims-data, indicators, amount of money
4 Procedures	Primary outcome: patients with acute respiratory infections (bronchitis, tonsillitis, sinusitis or otitis media), which are NOT treated with antibiotics.<br>The target value is empirically determined using claims-data (according to §295, §300 SGB V). Any practice reaching the target value will receive the additional reimbursement. This performance-based additional reimbursement is based on the total sum of the fund assigned in relation to the number of registered insured persons with index diseases in the practices.
5 Providers of intervention	The distribution key ('Verteilungsschlüssel') is defined by the consortium partners jointly.
6 Mode of delivery	Single intervention<br>All participating practices.
7 Location	The calculation of the outcomes is carried out by the aQua Institute and the payment of performance-based reimbursement is authorized by the Association of Statutory Health Insurance Physicians of Bavaria ('Kassenärztliche Vereinigung Bayerns').
8 Frequency	The service provision is provided in the quarters 2018q1 to 2019q2. There is a time shift in terms of additional reimbursement due to reasons of data provision and general reimbursement.
9 Planned tailoring	
10 Fidelity enhancement	

Component 6 E-learning on communication with patients for non-physician health professionals of the practice team

1 Short Name	E-learning for medical assistants to promote patient-centered communication.
2 Goal and rationale	The focus is on exploring patient expectations and shared decision-making in patients with non-complicated, acute infections, which are especially common in ambulatory practices and do not require the use of antibiotics as first choice treatment. The educational objective is to learn how to support the physician in his/her daily practice with patients with non-complicated infections and the rational use of antibiotics.
3 Materials	Video clips of re-enacted scenes of typical situations in the GP's daily practice, which are relevant for non-physician health professionals of the practice team.
4 Procedures	Various situations, typical for GP practices, are represented. In addition, the e-learning platform comprises background information of interest for medical assistants, which can be displayed by the users, if required.<br>The participation will be documented by an automatically generated personalized certificate.
5 Providers of intervention	The screenplay is produced at the Institute of General Medicine of the University of Rostock (and, if possible, involving MFAs) and implemented by the Society for Patient-centered Communication mbH (GPZK) in collaboration with a director and actors. The e-learning platform is established at the aQua Institute with the help of an external service provider.
6 Mode of delivery	Single intervention<br>Tailored for non-physician health professionals of the practice team from practices participating in intervention group B.
7 Location	The content is integrated into a Learning Management System (LMS). The learning platform will be accessible via a user-portal.<br>The learning contents on the learning platform are secured. Access for users can be provided via the specific user-portal. The learning contents are structured as self-learning courses. The learning platform will be available under the domain „welearn.academy“.<br>The correct functioning of video contents is ensured by an early test. The learning contents are generated in HTML5 format using the SCORM standards.
8 Frequency	Each course lasts about 1 hour and can be completed in the 4th quarter of 2017.
9 Planned tailoring	No
10 Fidelity enhancement	

Component 7 Quality circles with data-based feedback for non-physician health professionals of the practice team

1 Short Name	Quality circles with data-based feedback for medical assistants.
2 Goal and rationale	Self-reflection by means of practice-specific, data-based feedback reports, results of the patient survey and evidence-based background information combined with discussion and exchange of experiences in the quality circle together with other medical assistants.
3 Materials	Feedback reports tailored for medical assistants, based on claims-data (diagnosis and prescription data) and the results of the patient survey of one's own practice; checklists, patient information; slides for quality circle implementation.
4 Procedures	Claims-data (§§295, 300 SGB V) are provided by the participating health insurance companies pseudonymized for practices and anonymized for patient. Participating practices have signed a written informed consent to use their practice specific data, which means that specific claims-data can be allocated to each participating practice. A SFTP (Secure FILE Transfer Protocol) will be used for transmission of data between health insurance and the aQua Institute. Afterwards data will be checked and analyzed using SPSS and evaluated at the operations identification number (BSNR) level.<br>Individualized reporting is enabled by a self-developed software. The background information is guideline- and evidence-based (structured literature research) and relevant for non-physician health professionals of the practice team.
5 Providers of intervention	Physician, pharmacist, psychologist, IT-specialist, medical assistant
6 Mode of delivery	Individual and group intervention<br>Only for non-physician health professionals of the practice team from participating practices in intervention group B.
7 Location	The material will be sent by mail (practice-specific feedback) or will be available in the login area of the project portal (samples, slides) (arena-info.de).
8 Frequency	4x ca 2 hours in the quarters 2017q4, 2018q2+q4, 2019q2
9 Planned tailoring	
10 Fidelity enhancement	

Component 8 Information material available on tablet computers

1 Short Name	Information material on tablets
2 Goal and rationale	The information material focuses particularly on patients affected by infections and aim to raise awareness for the topic „antibiotics / resistance development”.
3 Materials	Topic-specific explanation videos and information on a tablet PC, multilingualism
4 Procedures	The core messages are guideline-based and developed in agreement with the other consortium partners. The material used are from recognized institutions, as well as self-generated ones in order to create a „Corporate Identity “.
5 Providers of intervention	Physician, pharmacist, psychologist, journalist, advertising expert.
6 Mode of delivery	Group intervention<br>Some of the material is also available via the portal.
7 Location	
8 Frequency	2017q4, 2018q1+q4, 2019q1+q2
9 Planned tailoring	
10 Fidelity enhancement	

Component 9 Computerized decision support system (CDSS)

1 Short Name	Computerized decision support system (CDSS).
2 Goal and rationale	Rational use of antibiotics; critical indication for non-complicated infections; reminders of targeted information for the patient which the physician should give and information on suitable other interventions (non-drug).
3 Materials	List of drug codes and diagnoses (reliable as well as suspected diagnoses) as well as prewritten reference texts.<br>Definition of certain case constellations for which reference texts will appear. Cooperation with software producers, specification of the so-called S3C interface; tool activation exclusively in the participating practices. These practices confirm the activation / use towards the aQua Institute.
4 Procedures	List of diagnosis and drug codes, in which certain instructions are available for the respective practice administration system. Writing of information texts.<br>The considered time interval (reference) is always the current quarter of a year. The following case constellations are defined (see figures):<br>*Scenario 1*<br>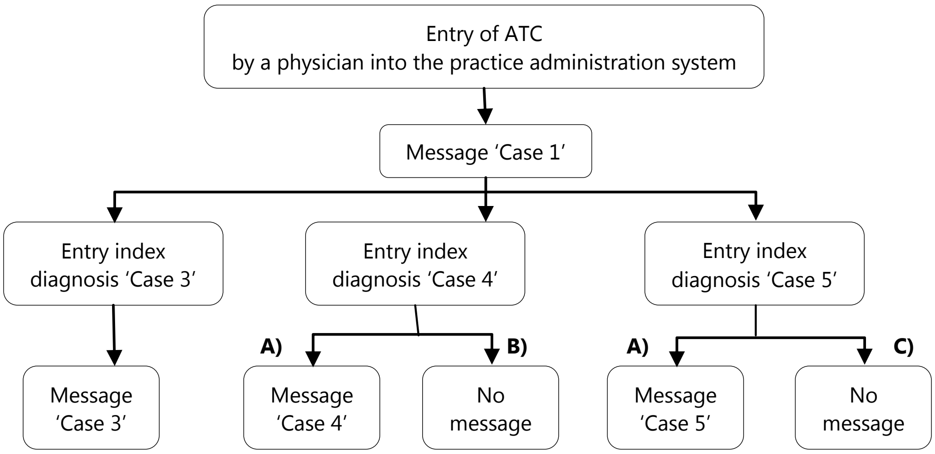<br><br>**A)** ATC = broad-spectrum antibiotics; **B)** ATC = first choice antibiotics; **C)** ATC = no broad-spectrum antibiotics

	Scenario 2<br>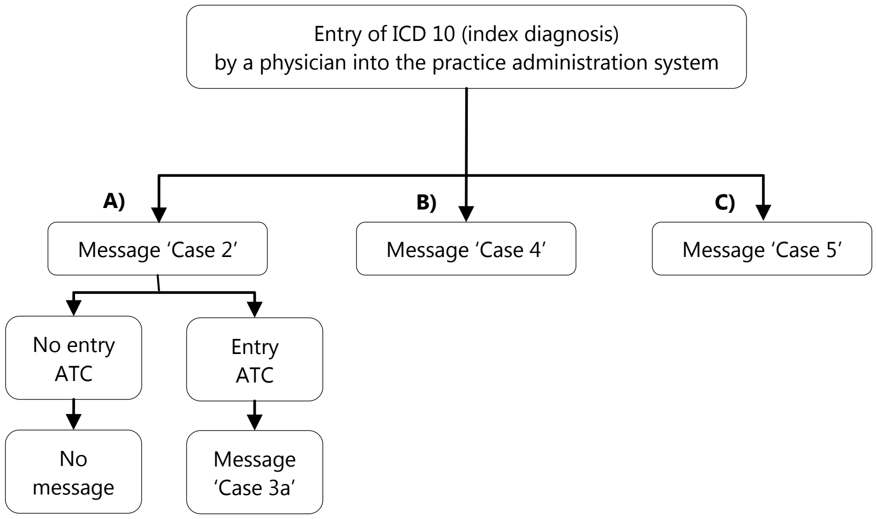<br><br>**A)** index diagnosis 'Case 2'; **B)** index diagnosis 'Case 4'; **C)** index diagnosis 'Case 5'
5 Providers of intervention	IT-specialist, physician, pharmacist.
6 Mode of delivery	Single intervention<br>Only for practices participating in intervention arm C.
7 Location	Definition of case constellations, in which instructions are given within the practice administration system. The specifications are made available in the form of Excel tables. These tables are the basis for the specifications of the so-called S3C interface by an external service provider.<br>This software tool will be integrated into the practice administration systems during a regular software update. An access restriction (release code) ensures that the IT decision support is only used by participating practices in intervention arm C.
8 Frequency	From 2018q3 to 2019q2
9 Planned tailoring	
10 Fidelity enhancement	

Component 10 Quality circles in local multidisciplinary groups

1 Short Name	Quality circles in local multidisciplinary groups.
2 Goal and rationale	Discussion and exchange of experience between different service providers.
3 Materials	Slides for the implementation of local multidisciplinary quality circles.
4 Procedures	The background information is guideline- and evidence-based (structured literature review). The care pathway of patients with non-complicated infections as well as the local situation on antimicrobial resistance is discussed. The meetings are moderated by GPs.
5 Providers of intervention	Physician, pharmacist, psychologist
6 Mode of delivery	Group interventions for healthcare providers in the geographic area covered by the participating networks in intervention arm C.
7 Location	The material are provided in the login area of the project portal (samples, slides) (arena-info.de).
8 Frequency	2x ca 2 hours in the quarters 2018q1+q2 and 2018q4 and 2019q1 respectively
9 Planned tailoring	
10 Fidelity enhancement	
