# Supplementary material for: Sustainable reduction of antibiotic-induced antimicrobial resistance (ARena) in German ambulatory care: study protocol of a cluster randomised trial
Source: Implement Sci. 2018 Feb 5;13:23. doi: 10.1186/s13012-018-0722-0 (PMC5800289; doi:10.1186/s13012-018-0722-0)
Supplement: Supplementary file 2 — Eligible medical specialist groups for participation in the ARena study (Table S1); description of the recruitment of participants in the ARena study (Table S2). (PDF 222 kb) [file 13012_2018_722_MOESM2_ESM.pdf]

Additional file 2

Table 1 Eligible medical specialist groups for participation in the ARena study

**Medical specialist group**	**German equivalent** (Facharztgruppe-FG)
General practitioner (GP)	Allgemeinmediziner, praktischer Arzt, hausärztlich tätige Internist (FG 01, 02, 03)
Internist	Internist (FG 23)
Gynecologist	Gynäkologe (FG 15)
Ear, nose and throat (ENT) specialist	Hals-Nasen-Ohren (HNO) Arzt (FG 19)
Urologist	Urologe (FG 67)
Pulmonary specialist	Pneumologe (FG 30)
Pediatrician	Kinderarzt (FG 34, 46)

Table 2 Description of the recruitment of participants in the ARena study

	**Recruitment strategy**
General study participation / outcome evaluation	In total, 221 ambulatory practices (302 physicians, respectively) in the fourteen practice networks will be invited to participate in the study (arm A: 68 practices / 102 physicians; arm B: 56 practices / 91 physicians; arm C 68 practices / 112 physicians). All practices will be included in the claims-data based outcome evaluation. The comparison group will be selected randomly from ambulatory practices outside of the participating networks. Additionally, research-practices of the Dept. of General Practices and Health Services Research will be invited for participation in the patient survey pilot study.
Patient survey	Per practice (in study arm B) 60 patients aged 18 years or above and insured at the AOK health insurance in Bavaria with index diseases will be asked by their physician to participate (anonymously and voluntarily) in the patients survey by filling in the questionnaire (main study). The pilot study is conducted in practices which do not take part in the ARena main study (10-12 practices, 10-15 patients per practice). In the pilot study one or two patients per practice are asked by their doctor, in addition to filling in the questionnaire, to participate in a short telephone interview conducted by a researcher. All patients receive an information leaflet concerning the aim and the background of the survey, including information on data protection.
Process evaluation	Physicians in all three intervention arms and non-physician health professionals of the practice team (medical assistants) in intervention arm B, who were contacted for general study participation, already received information about the process evaluation survey through their information leaflet.<br>Selection of potential interview partners within the sample of physicians and medical assistants willing to participate in the intervention study, and stakeholders will be based on the strategy of purposive sampling. This sampling technique helps to identify individuals that are especially knowledgeable about and/or experienced in the phenomenon of interest.<br>Every participating practice network is asked to name a GP, who is involved in the ARena-interventions and who is interested to take part in the focus group.
